# Pedicled versus skeletonized internal thoracic artery grafts: a randomized trial

**DOI:** 10.1177/0218492320983491

**Published:** 2020-12-17

**Authors:** Mats Dreifaldt, Ninos Samano, Håkan Geijer, Mats Lidén, Lennart Bodin, Domingos Souza

**Affiliations:** 1 Department of Cardiovascular and Thoracic Surgery, School of Medical Sciences, Örebro University, Örebro, Sweden; 2 Department of Cardiothoracic and Vascular Surgery and University Health Care Research Center, Faculty of Medicine and Health, Örebro University, Örebro, Sweden; 3 Department of Radiology, School of Medical Sciences, Örebro University, Örebro, Sweden; 4 Institute of Environmental Medicine; Unit of Intervention and Implementation Research, Karolinska Institute, Stockholm, Sweden

**Keywords:** Coronary artery bypass, graft occlusion, saphenous vein, thoracic arteries, tissue

## Abstract

**Objective:**

Concerns have been raised regarding whether skeletonization of the internal thoracic artery could damage the graft and thereby reduces its patency. The objective of this study was to compare patency rates at mid- and long-term follow-up between pedicled and skeletonized left internal thoracic artery grafts.

**Methods:**

This randomized controlled trial included 109 patients undergoing coronary artery bypass surgery. The patients were assigned to receive either one pedicled or one skeletonized left internal thoracic artery graft to the left anterior descending artery. Follow-up was performed at 3 years with conventional angiography, and at 8 years with computed tomography angiography. Differences between patency rates were analyzed with Fisher’s exact test and a generalized linear model.

**Results:**

The patency rates for pedicled and skeletonized left internal thoracic artery grafts were 46/48 (95.8%) versus 47/52 (90.4%), *p* = 0.44 at 3 years, and 40/43 (93.0%) versus 37/41 (90.2%), *p* = 0.71 at 8 years, respectively. The difference in patency rates for pedicled and skeletonized grafts was 5.4% (95% confidence interval: −4.2–14.5) at 3 years and 2.8% (95% confidence interval: −9.9–14.1) at 8 years. All failed grafts, except for one with a localized stenosis, were anastomosed to native coronary arteries with a stenosis less than 70%. Three patients suffered sternal wound infections (two in the pedicled group, one in the skeletonized group).

**Conclusions:**

The skeletonization technique can be used without jeopardizing the patency of the left internal thoracic artery. The most important factor in graft failure was target artery stenosis below 70%.

## Introduction

The left internal thoracic artery (LITA) is the graft of choice in coronary artery bypass grafting (CABG). This is based on convincing evidence of improved survival in patients with critical left anterior descending artery (LAD) disease in whom an LITA graft has been placed.^1^ The long-term patency of the LITA is approximately 90% 10 years after CABG.^2^ Bilateral internal thoracic arteries (BITA) are used in a low percentage of CABG operations despite excellent results.^3^ Patients who received BITA grafts for left coronary system revascularization have improved early and late outcomes and decreased risk of death, reoperation, and additional reinterventions.^4^ Most commonly, the LITA is harvested as a pedicled graft and used to bypass the LAD. However, the LITA can also be harvested in a skeletonized fashion, especially when using the BITA approach, reducing trauma and postoperative sternal complications.^5^ Advocates of skeletonization also emphasize that it increases the number of arterial anastomoses per patient by increasing the length of the LITA and thereby making it possible to perform sequential grafting. There are a few randomized trials comparing outcomes between pedicled LITA (P-LITA) and skeletonized LITA (S-LITA),^6^ and to our knowledge, there is no randomized trial comparing long-term patency between these two harvesting techniques. Consequently, in 2003 we started a prospective randomized trial with the aim of comparing patency rates between P-LITA and S-LITA at both mid- and long-term follow-up.

## Patients and methods

This single-center randomized trial studied consecutive patients undergoing elective, first-time, isolated CABG surgery. The study was approved by the local ethics committee, and written informed consent was obtained from each participant. Between January 2004 and August 2009, 109 patients were included in the study.

This randomized trial was designed mainly to compare the patency rates between radial artery (RA) and no-touch (NT) saphenous vein (SV) grafts. The LITA was scheduled to bypass the LAD and was in a separate procedure randomized to be used either as a pedicled or skeletonized conduit, while the RA and NT SV grafts were used as complementary grafts to bypass the left and right coronary territories. The mid- and long-term results of the RA vs. NT SV trial have been published previously.^7^,^8^ Patients who had at least three-vessel coronary artery disease were eligible for inclusion. Exclusion criteria were age > 65 years, left ventricular ejection fraction < 40%, serum creatinine > 120 μmol·L^−1^, use of anticoagulants, coagulopathy, allergy to contrast medium, a positive Allen’s test or an abnormal Doppler study of the arms, history of vasculitis or Raynaud’s syndrome, bilateral varicose veins, or previous vein stripping. Acetylsalicylic acid 150 mg (Pfizer, Inc., New York, USA) was administered within six hours postoperatively; thereafter, 75 mg was prescribed daily. Calcium channel blockers were only used for treatment of hypertension.

Computer-generated block randomization was used. The surgeon enrolled and assigned participants to the intervention. The randomization was revealed in the operating room by opening numbered sealed envelopes provided by the statistician. Angiography assessors were independent and blinded to the outcome of randomization.

All patients were operated on-pump by the same surgical team. P-LITA was harvested using electrical cautery and titanium clips. S-LITA was harvested in situ using sharp dissection and titanium clips. When dissection was completed, the LITAs in both groups were left in situ until after heparinization, embedded in a 1 mg·mL^−1^ papaverine-soaked sponge. Prior to extracorporeal circulation, the grafts were distally divided and free flow was measured. If the free flow was less than 50 mL·min^−1^, papaverine was injected intraluminally. The distal end was then sealed with a clip and the graft was left imbedded in the papaverine-soaked sponge until use. The classic principle of revascularization was followed, using the LITA to bypass any LAD with a stenosis greater than 50%. Calibrated probes were used to measure the diameters of grafted coronary arteries. In both groups, the LITA anastomoses were performed using 8/0 Prolene continuous sutures. Transient time flow measurements of the grafts were performed after weaning from extracorporeal circulation once stable hemodynamic conditions were achieved, using an ultrasonic transit-time flowmeter (VeriQ system, Medi-Stim, Inc., USA).

Conventional angiographies were performed using Philips angiographic equipment (Integris H3000, Philips, Netherlands) with manual injections of Iodixanol 320 mg·mL^−1^ (Visipaque, GE Healthcare) at mean time of 3 years postoperatively. In cases of suspected spasm, nitroglycerine was injected. The angiograms included at least two orthogonal views of each graft (45° left anterior oblique and 45° right anterior oblique). The LITA grafts were categorized as either patent or failed. Failed grafts were defined as having a stenosis more than 70% of the diameter in any part of the graft or the presence of a string sign (diffuse narrowing of the graft to less than 1 mm in diameter with persistent flow).

Computed tomography (CT) angiography with a Somatom Flash dual-source CT scanner (Siemens, Erlangen, Germany) was the assessment of choice at a mean time of 8 years postoperatively. All subjects received nitroglycerin 0.25 mg sublingually, and those with a heart rate > 70 beats·min^−1^ and no contraindications were also given up to 10 mg of metoprolol intravenously before the examination. Contrast medium (60–70 mL of Iomeron 400 mg·mL^−1^, Bracco, Milan, Italy) was administered with a pressure injector at a flow rate of 6 mL·s^−1^, followed by a bolus of 60 mL of saline. Scanning started at the left subclavian artery and ended at the base of the heart. The images were reviewed at a Siemens SyngoVia workstation. All images were independently reviewed by two thoracic radiologists who were blinded to group assignment. Disagreements were resolved by consensus. Where possible, the studies were compared with reports and images from previous coronary angiographies. A graft was judged to be occluded when it was not opacified by contrast medium. A graft stenosis was judged to be significant when narrowing of the lumen diameter was > 50% relative to the adjacent parts of the vessel.

As this was a sub-study, the sample size was calculated based on the main study that included 109 patients. All of these patients were included with random allocation of the grafts to either skeletonization or non-skeletonization. Difference between patency in the two randomized groups was analyzed with Fisher’s exact test, using a computational algorithm for small and skewed distributions. To obtain a reliable confidence interval (CI) for the central effect parameter, that is, the difference in patency rates, to supplement the calculation of *p* values, a generalized linear model was used. The outcome in this model was thus the difference in patency rates (p_1_ – p_2_) and the analysis provided an estimate of this difference and its 95%CI. To comply with statistical assumptions for the CI, a bootstrap calculation of the standard error of the CI based on 1000 independent replications was used. With the linear model, it was also possible to introduce potential confounders and interaction factors in the analysis, but in the final analytical model, no additional covariates were used because they introduced only very small changes in the effect parameter, the difference in patency. For differences between the two groups regarding continuous outcome variables such as graft flow, Student’s *t* test was used. Agreement between images was estimated by the kappa and weighted kappa method. The criterion for statistical significance was *p* less than 0.05. Statistical software (SPSS version 25 and STATA version 15) was used for analyses.

## Results

**Figure 1.**
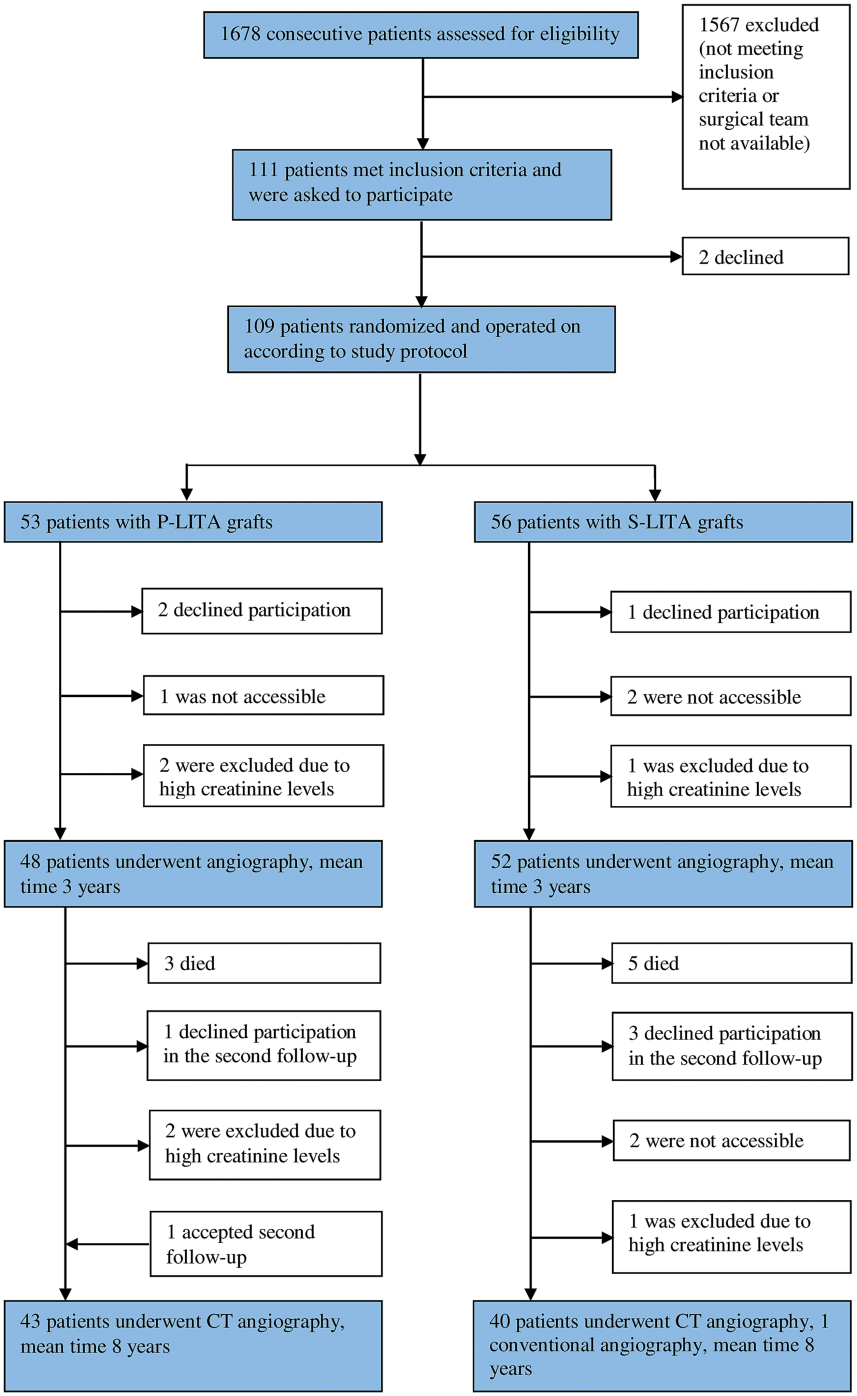
Consort flow diagram of patient inclusion and follow-up at 3 and 8 years. CT: computed tomography; P-LITA: pedicled left internal thoracic artery; S-LITA: skeletonized left internal thoracic artery.

The first follow-up was performed between March 2009 and November 2010, at a mean time of 3 years (range 1–5.8 years) after surgery, and the second follow-up between October 2014 and May 2015, at a mean time of 8 years (range 5.5–10.7 years) after surgery. The consort flow chart is illustrated in Figure 1. Two eligible male patients, aged 61 and 64 years, declined participation in the study. Patient characteristics of the study cohorts are presented in Table 1. No deaths occurred perioperatively or within 3 years. At 8 years, 8 patients had died (3 P-LITA and 5 S-LITA). None of the patients followed-up had a new myocardial infarction within 3 years but one with an occluded P-LITA developed a new myocardial infarction within 8 years. At 8 years, 2 patients (both S-LITA, both female) had undergone percutaneous coronary interventions directed towards the LAD. The majority of patients used acetylsalicylic acid 75 mg daily at the 3-year and 8-year follow-up, 92% and 87% respectively; none were on dual antiplatelet therapy. At 3 years, 23 (23%) patients were on calcium channel blockers, and 20 (24%) at 8 years. Three patients suffered sternal wound infections (2 P-LITA and 1 S-LITA). In 3 patients, the LITA was considered unsuitable for use. In 2 patients (both S-LITA), a single NT SV graft was used as a substitute for the LITA, and in one (P-LITA), the LAD was bypassed side to side with a triple sequential NT SV graft to the left territory. All grafts were analyzed according to the intention-to-treat principle. Free flow < 50 mL·min^−1^ was found in 19/47 (40%) patients in the P-LITA group and 27/50 (54%) in the S-LITA group and was treated with intraluminal instillation of papaverine. After 3 years, 6/7 failed grafts, and 5/7 after 8 years, had free flow < 50 mL·min^−1^ initially. The mean graft flow after weaning from extracorporeal circulation did not differ (*p* = 0.8) between the P-LITA (41.7 mL·min^−1^) and S-LITA (41.9 mL·min^−1^) groups. Table 2 shows the differences in patency rates. The characteristics of the 100 LITA grafts 3 years postoperatively and subgroup analysis are presented in Table 3. Seven of eight failed grafts, including one S-LITA that showed a string sign, were anastomosed to an LAD with <70% stenosis; 5 had been treated with papaverine. The patency rates for grafts anastomosed to a target vessel with <70% stenosis were P-LITA (21/23, 91%) vs. S-LITA (14/18, 78%). All SV grafts to the LAD were patent. The consensus between the two angiography assessors was high. Regarding patent grafts, the agreement was excellent, kappa = 1.0; and for native coronary artery stenosis preoperatively, the weighted kappa value was 0.70 (95%CI: 0.58–0.82). The characteristics of the 84 LITA grafts 8 years postoperatively and subgroup analysis are presented in Table 3. Two P-LITA and one S-LITA that were patent at 3 years were occluded at 8 years. One S-LITA that showed a string sign at 3 years was patent at 8 years. All failed LITA grafts, except one with a localized stenosis (S-LITA), were connected to an LAD with <70% stenosis. Further analysis of LITA patency within each group revealed that the patency rate of S-LITA grafts anastomosed to an LAD with <70% stenosis was significantly lower than that of S-LITA to LAD targets with ≥70% stenosis. This was true at both follow-up periods. The same was not seen when analyzing the patency differences within the P-LITA group (Table 4). All 3 SV grafts that were substitutes for LITA (1 P-LITA and 2 S-LITA) were patent. One patient who did not participate in the first follow-up showed a patent P-LITA at 8 years.

**Table 1.** Patient characteristics at 3- and 8-year follow-up.

	3 years		8 years	
Variable	P-LITA (*n* = 48)	S-LITA (*n* = 52)	P-LITA (*n* = 43)	S-LITA (*n* = 41)
Age (years) [range]	58.9 ± 5.4 [40–65]	59.6 ± 5.8 [39–65]	59.0 ± 5.6 [40–64]	59.1 ± 6.7 [41–65]
Myocardial infarction	16 (33%)	18 (35%)	13 (30%)	14 (34%)
Female	8 (17%)	4 (8%)	7 (16%)	3 (7%)
NYHA class				
I	1 (2%)	3 (6%)	1 (2%)	2 (5%)
II	17 (35%)	18 (35%)	17 (40%)	14 (34%)
III	25 (52%)	23 (44%)	20 (47%)	21 (51%)
IV	5 (10%)	8 (15%)	5 (11%)	4 (10%)
Diabetes	9 (19%)	8 (15%)	8 (19%)	5 (12%)
Hypertension	26 (54%)	24 (46%)	26 (60%)	19 (46%)
Never smoked	18 (38%)	21 (40%)	15 (35%)	18 (44%)
Ex-smoker	21 (44%)	16 (31%)	19 (44%)	16 (39%)
Current smoker (1 year)	9 (19%)	15 (29%)	5 (12%)	8 (20%)

NYHA: New York Heart Association; P-LITA: pedicled left internal thoracic artery; S-LITA: skeletonized left internal thoracic artery.

**Table 2.** Difference in patency rates between P-LITA and S-LITA at 3 and 8 years.

Variable	P-LITA		S-LITA		P-LITA-S-LITA		
Time after surgery (years)	Assessed	Patent	Assessed	Patent	Difference in patency	95%CI	*p* value
3	48	46 (95.8%)	52	47 (90.4%)	5.4	−4.2%–14.5%	0.44
8	43	40 (93.0%)	41	37 (90.2%)	2.8	−9.9%–14.1%	0.71

CI: confidence interval; P-LITA: pedicled left internal thoracic artery; S-LITA: skeletonized left internal thoracic artery.

**Table 3.** Subgroup analysis: patency rates according to the grafts and target coronary artery characteristics at 3 and 8 years.

	3 years				8 years			
	P-LITA		S-LITA		P-LITA		S-LITA	
	Assessed	Patent	Assessed	Patent	Assessed	Patent	Assessed	Patent
Free ITA flow								
<50 mL·min^−1^	19	17 (89%)	27	23 (85%)	14	12 (86%)	23	20 (87%)
≥50 mL·min^−1^	28	28 (100%)	23	22 (95%)	28	27 (96%)	16	15 (94%)
ITA flow								
<20 mL·min^−1^	12	12 (100%)	2	2 (100%)	10	9 (90%)	2	2 (100%)
20–39 mL·min^−1^	13	12 (92%)	26	22 (100%)	13	12 (92%)	19	16 (84%)
40–60 mL·min^−1^	12	12 (100%)	15	15 (100%)	10	9 (90%)	13	13 (100%)
60 mL·min^−1^	9	9 (100%)	7	6 (86%)	9	9 (100%)	4	4 (100%)
LAD stenosis								
<70%	23	21 (91%)	18	14 (78%)	20	17 (85%)	15	12 (80%)
70–89%	16	16 (100%)	17	16 (95%)	13	13 (100%)	10	10 (100%)
90–100%	9	9 (100%)	17	17 (100%)	9	9 (100%)	14	14 (100%)
LAD size								
TEA	1	1 (100%)	1	1 (100%)	1	1 (100%)	1	1 (100%)
<1.5 mm	14	14 (100%)	14	14 (100%)	10	9 (90%)	14	12 (86%)
1.5–2.0 mm	22	21 (96%)	27	24 (89%)	23	21 (91%)	17	16 (94%)
>2.0 mm	11	10 (91%)	10	8 (80%)	9	9 (100%)	9	8 (89%)
LAD quality								
Good	39	37 (95%)	39	35 (90%)	34	32 (94%)	29	27 (93%)
Mild calcification	6	6 (100%)	7	6 (86%)	6	6 (100%)	5	4 (80%)
Severe calcification	3	3 (100%)	6	6 (100%)	3	2 (67%)	7	6 (86%)

ITA: internal thoracic artery; LAD: left anterior descending artery; P-LITA: pedicled left internal thoracic artery; S-LITA: skeletonized left internal thoracic artery; TEA: thromboendarterectomy.

**Table 4.** Patency rates according to degree of artery stenosis and in pedicled and skeletonized left internal thoracic artery at 3 and 8 years.

	3 years				8 years				
	P-LITA		S-LITA		P-LITA			S-LITA	
LAD stenosis	Patent	*p* value, LAD <70% vs. ≥70%	Patent	*p* value, LAD <70% vs. ≥70%	Patent	*p* value, LAD <70% vs. ≥70%		Patent	*p* value, LAD <70% vs. ≥70%
<70%	21/23		14/18		17/20			12/15	
		0.224		0.043		0.099			0.050
≥70%	25/25		33/34		22/22			24/24	

	Stenosis < 70%		Stenosis ≥70%		Stenosis < 70%			Stenosis ≥70%	
	Patent	*p* value, P-LITA vs. S-LITA	Patent	*p* value, P-LITA vs. S-LITA	Patent		*p* value, P-LITA vs. S-LITA	Patent	*p* value, P-LITA vs. S-LITA
P-LITA	21/23		25/25		17/20			22/22	
		0.377		1.000			1.000		
S-LITA	14/18		33/34		12/15			24/24	

LAD: left anterior descending artery; P-LITA: pedicled left internal thoracic artery; S-LITA: skeletonized left internal thoracic artery.

## Discussion

To our knowledge, this is the first randomized longitudinal study comparing mid- and long-term patency rates between P-LITA and S-LITA. The patency rates at mean times of 3 and 8 years after surgery were excellent for both P-LITA and S-LITA; 95.8% and 90.4% at 3 years with only somewhat lower rates, 93.0% and 90.2%, at 8 years. This study thus showed that both P-LITA and S-LITA provide excellent mid-and long-term graft patency rates, demonstrating that the harvesting technique did not play any significant role in the patency of the LITA. Of particular note is that all failed LITA grafts, except one with a localized stenosis, were anastomosed to an LAD with <70% stenosis. This is in keeping with recent data by Harskamp and colleagues^9^ showing that lower-grade stenosis of the LAD was one of the strongest predictors of LITA failure as a consequence of excessive competitive flow. Further analysis showed a significantly lower patency rate for S-LITA anastomosed to an LAD with <70% stenosis compared to S-LITA anastomosed to an LAD with ≥70% stenosis. Although interesting, this should be cautiously interpreted due to the low number of grafts within each group.

The concept of skeletonization of the ITA is not new. The technique was described in detail by Keeley^10^ in 1987. Harvesting of a skeletonized ITA is more time-consuming and requires a higher level of surgical precision compared to pedicled conduits. It has been shown that harvesting the ITA as a pedicled graft reduces blood supply to the sternum compared to skeletonized grafts.^11^ There are studies showing that S-LITA appears to reduce the incidence of postoperative sternal wound infections compared to P-LITA after CABG.^12^ However, the latest publication from the ART trial did not show any strong benefit of skeletonization of a single ITA but was beneficial when using BITA, particularly in high-risk patients.^5^

The question has been raised of whether the skeletonization technique may cause damage to the ITA vessel wall. It has been suggested that depriving the ITA of vasa vasorum, innervation, and lymphatic and venous drainage by skeletonization, together with creating an imbalance between vasoconstriction and vasodilation substances, supports the superiority of pedicled grafts.^13^ However, in contrast to the SV, the ITA is unlikely to suffer from ischemia if the vasa vasorum is disrupted by skeletonization because the vasa vasorum is significantly smaller in the ITA compared to SV grafts.^14^ Furthermore, the functional integrity of skeletonized ITA has been shown to be similar to that of pedicled ITA in both acute and chronic phases. Although skeletonization induces neovascularization in the adventitia, it does not induce proliferation of smooth muscle cells in the media, which is reported to be a feature of vascular remodeling. Gaudino and colleagues^15^ reported that skeletonization does not impair the vasoactive profile of S-LITA grafts. Additionally, several studies have shown excellent patency rates for skeletonized ITA.^16^ Moreover, some reports showed that skeletonized RA grafts have a higher patency rate than pedicled RA grafts and that skeletonized gastroepiploic artery grafts also have excellent patency rates. Contrary to some studies,^17^ but in accordance with others,^6^ we could not demonstrate any difference in post-anastomotic graft flow between P-LITA and S-LITA grafts.

On the other hand, skeletonization of the SV seems to have a negative effect on long-term patency rates,^18^,^19^ and that harvesting the SV with a pedicle, NT technique, produces higher patency rates than that for pedicled radial artery grafts.^7^,^8^ A number of factors may explain the improved performance of NT SV grafts,^20^ such as providing an intact endothelium and the preservation of vasa vasorum that may prevent medial ischemia. The avoidance of adventitial activation by surgical trauma may prevent medial and intimal myofibroblast infiltration, and retention of the adventitial vasa vasorum and surrounding fat may have beneficial paracrine effects on vein graft smooth muscle.^20^ This suggests that the skeletonization harvesting technique is more detrimental to SV than arterial grafts.

This was a randomized study with follow-up of up to 8 years. This study together with our previous experience suggests that preservation of the surrounding fat tissue is more important for SV rather than for arterial grafts. The major limitation is the sample size, due to being a sub-study to the main study that compared NT SV grafts with RA grafts. Therefore, this sub-study is underpowered to show statistical equivalence for small differences in graft patencies. Less than 10% of these patients were included in the original study. The exclusion criteria, especially age < 65 years and left ventricular ejection fraction < 40% resulted in excluding almost 2/3 of patients. The other criteria including unavailability of the surgical team resulted in excluding the other 1/3. This is a possible source of selection bias. In addition, it was a single-center study in which P-LITA is the standard and S-LITA is seldom used. Even though the surgeons who performed these procedures were very experienced, P-LITA had the advantage from this perspective.

We concluded that the patency rates of both P-LITA and S-LITA are excellent at both mid- and long-term in patients aged 65 years or younger. The most important factor in LITA failure was the degree of stenosis of the LAD and not the surgical technique used for graft preparation. A larger randomized multicenter study is required to confirm the results.

Registration: ClinicalTrials.gov: NCT02158455.
